# Persistent hiccup reflex activation as a complication of dental implant surgery: a case report

**DOI:** 10.1093/omcr/omy027

**Published:** 2018-06-25

**Authors:** Gianluca Porcaro, Lucio Tremolizzo, Ildebrando Appollonio, Marcello Maddalone

**Affiliations:** 1 Dental Unit, ‘San Gerardo’ Hospital and School of Medicine and Surgery, University of Milano-Bicocca, Monza, Italy; 2 Neurology Unit, ‘San Gerardo’ Hospital and School of Medicine and Surgery, University of Milano-Bicocca, Monza, Italy

## Abstract

Persistent hiccup can conceal life-threatening clinical conditions of highly heterogeneous nature. Here, we report a case of a persistent hiccup that has been temporally associated with dental implant insertion in a patient with paroxysmal atrial fibrillation. This 67-year-old patient underwent dental implant in area 36 and the procedure was performed without acute complications. A prolonged hiccup started ~48 h after the procedure: it failed to respond to several medications and it lasted for ~18 days, after which it spontaneously disappeared. Brain imaging and neurological examination were conducted in order to rule out organic conditions, even though all the investigations resulted to be negative. We suggest that the surgical procedure might have triggered a prolonged reflex activation. Knowledge about this complication of oral surgery procedure might be useful for avoiding unnecessary hospitalization or diagnostic tests.

## INTRODUCTION

Hiccup, or ‘singultus’, i.e. the phenomenon of repeated spontaneous myoclonic contractions of the diaphragm, is usually a self-limiting disorder, mostly lacking a specific clinical relevance [1, 2]. However, ‘persistent’ hiccup—defined as a singultus that lasts from 48 h to 1 month—may produce a significant discomfort and it may also represent the clinical manifestation of life-threatening clinical conditions such as lateral medullary lesions, myocardial infarction and pulmonary embolism [1, 2]. Both central and peripheral nervous system lesions can induce a persistent hiccup by interfering, respectively, with either the afferent or the efferent fibers linked to respiratory muscles, or the integration centers located within the brainstem, especially the vagal nuclei and the nucleus tractus solitarius located in the medulla oblongata. Medical conditions affecting the esophagus and other parts of the gastrointestinal tract are also associated with protracted hiccups, due to stimulation of the visceral afferent fibers of the vagus nerve. Moreover, metabolic and endocrine disorders, drugs, general anesthesia and psychogenic problems may also cause hiccup. The exact involved mechanisms are not completely clear, and Nardone *et al*. [3] report as many authors consider hiccup the subcortical equivalent of myoclonus generated at the pontomedullary level, whereas other consider it related to denervation supersensitivity caused by dysfunction of the inferior olivary complex, nucleus ambiguous and adjacent reticular formation of the medulla oblongata [3, 4].

Here, we report the case of a persistent hiccup following an oral surgery procedure, briefly discussing the potential mechanisms which might have triggered a prolonged reflex activation.

## CASE PRESENTATION

A 67-year-old Caucasian man came to our attention after a fixed oral prosthesis surgery. His past medical history was significant for a paroxysmal atrial fibrillation for which he was taking Amiodarone and Acenocumarol with stable International Normalized Ratio (INR) values. Following orthopantomography and CT scan, the procedure of implant insertion was performed with local anesthesia (Mepivacaine and vasoconstrictor) in area 36 [left lower jaw, designated according to the ISO system] [5] without any complication. Acenocumarol had been discontinued 2 days before, switching to LMW heparin. The patient was discharged under antibiotic therapy (Amoxicillin and Clavulanic acid) and instructed to restart oral anticoagulant therapy after 2 days. About 48 h after the procedure, hiccup abruptly presented and failed to cease.

The patient did not report any further symptom, and no complications were found at the revision of the surgical area. Three days after the onset, the patient came to the emergency department due to this persisting symptom: Baclofen, Metoclopramide and Bromazepam were administered without significant clinical improvement. Both ENT, neurological examinations, blood tests and a brain CT scan failed to show any abnormality. Three days later, Chlorpromazine 25 mg b.i.d. was administered for 2 weeks. Furthermore, the patient was advised to program in the next few days a brain MR scan and chest imaging that were negative. Seven days following the surgical procedure, the stitches were removed and the wound did not show any problem. The hiccup continued resulting in significant distress and sleep deprivation. Providentially, it spontaneously ceased after 18 days. Neither relapses or neurological symptoms were reported in the later months.

## DISCUSSION

Although uncommon, it has already been reported that oral surgery procedures may be potentially complicated by persistent hiccup [6, 7]. We hypothesized that the hiccup affecting our patient could have been generated by the stimulation of peripheral receptors projecting to the intermediate part of the spinal tract nucleus of the fifth cranial nerve, leading to the activation of fibers belonging to the phrenic nucleus and the nucleus ambiguous [1]. Moreover, cases of abrupt hiccup in healthy subjects after chin shaving or stroking have been documented, supporting our hypothesis [8]. The delayed onset of the hiccup could apparently refute the hypothesis of afferent nerve fibers stimulation, even though the same temporal pattern has already been shown in similar conditions [6]. Other peripheral triggers were excluded in our case by a careful oral and ENT examination, besides other potential concomitant conditions arising at the mediastinum level. In particular, gastric distension is a very common cause [2], but our patient specifically denied any gastrointestinal symptom.

General anesthesia procedures can be complicated by persistent hiccup, which has been at least in part attributed to the disinhibition of phrenic nerve motor drives [1]. However, this condition does not apply here, since only local anesthesia was employed. Albeit we regard any iatrogenic contribution as probably not influential, exposure to some antibiotics (e.g. macrolides) has been reported among rare causes of persistent hiccup [2]. Similarly, our patient was not exposed to drugs such as benzodiazepines or steroids, or to chemo-radiotherapy that are known causes of such phenomenon [3]. In our case we had to carefully consider causes unrelated to central nervous system (CNS), since non-CNS triggers have been reported with significantly increased frequency in male patients with respect to female ones [9].

Regarding CNS-related causes, the change of the anticoagulant scheme in our patient determined a potential risk factor for acute cerebrovascular accidents of embolic origin [10], but brain imaging excluded both brain hemorrhages and ischemic lesions in the posterior fossa. Moreover, the presence of an isolated hiccup without any further neurological deficit did not strongly support the hypothesis of a cerebrovascular event, since most cases of lateral medullary infarctions present with multiple additional neurological symptoms, and sometimes even include non-specific ones, such as headache, nausea or neck pain [10–12]. In partial contrast with this affirmation, a case of isolated hiccup due to supratentorial brain infarct has been actually reported [13]. Furthermore, several neurological conditions leading to the development of a persistent hiccup have been reported as well [11, 14, 15].

In summary, we consider the involuntary stimulation of the hiccup afferent pathway as the most probable predisposing factor for this prolonged activation. Obviously, albeit implausible, a putative psychogenic origin was not ruled out, and the strict temporal association between the surgical procedure and the onset of persistent hiccup would have just a circumstantial value. Nevertheless, we believe that both the oral surgery specialist and the neurologist should be aware of this rare but extremely distressing complication, in order to avoid unnecessary ED admissions and diagnostic tests.
